# Validation of a quantitative web-based food frequency questionnaire to assess dietary intake in the adult Emirati population

**DOI:** 10.1371/journal.pone.0262150

**Published:** 2022-01-27

**Authors:** Najoua El Mesmoudi, Ayesha S. Al Dhaheri, Jack Feehan, Lily Stojanovska, Habiba I. Ali

**Affiliations:** 1 Department of Nutrition and Health, College of Medicine and Health Sciences, United Arab Emirates University, Al Ain, United Arab Emirates; 2 The Department of Medicine–Western Health, The University of Melbourne, Melbourne, Australia; 3 The Institute for Health and Sport, Victoria University, Melbourne Australia; Higher Institute of Applied Sciences and Technology of Gabes University of Gabes, TUNISIA

## Abstract

**Background and objective:**

A culture-specific web-based food frequency questionnaire (FFQ) to assess dietary intake in the United Arab Emirates (UAE) adult population was developed using data from the 2009–2010 national nutrition survey. The objective of this study was to assess the relative validity of the newly developed FFQ for use in the adult Emirati population (AE-FFQ), which contained a list of 139 food lines.

**Methods:**

A convenient sample of 60 (36 females and 24 males) adult Emiratis completed 3 non-consecutive 24HRs over a period of one month, followed by the AE-FFQ, which assessed the intake over the previous month. Relative validity was evaluated by comparing nutrient and food group intakes from the AE-FFQ with the average three 24HRs using Wilcoxon signed-rank tests, Spearman’s correlation coefficients (CC), Bland-Altman analysis, and cross-classification.

**Results:**

The AE-FFQ overestimated energy and most nutrients and food groups. Bland–Altman analysis showed significant proportional bias between the 2 methods. Deattenuated energy-adjusted Spearman correlation coefficients were poor to good ranging from 0.06 (iron) to 0.62 (fiber) for nutrients, 0.39 median value, and from –0.01 (cruciferous vegetables) to 0.64 (eggs) for food groups, 0.41 median value. A fairly acceptable agreement was obtained, with correct classification into the same or adjacent quartile ranging from 34% (vitamin B12) to 78% (pyridoxine), median 69% for nutrients and from 55% (diet soft drinks) to 87% (soft drinks), median 67% for food groups.

**Conclusions:**

The AE-FFQ is an acceptable tool for ranking UAE adults (aged 18 to 50) according to their dietary intake to investigate the role of Emirati dietary patterns on health and disease. Caution is needed for assessing absolute intake, however, given the bias observed in assessing group-level agreement.

## Background

Suboptimal diet (i.e., high in sodium and low in whole grains, fruits, nuts & seeds, vegetables and omega-3 fatty acids) is well established as one of the most important modifiable risk factors of noncommunicable disease, including obesity, cardiovascular disease (CVD), type 2 diabetes, and certain cancers [1]. Unhealthy eating habits have been shown to cause about 11 million deaths globally and 255 million disability-adjusted life-years (DALYs, a measure of global burden of disease), making suboptimal diet the leading cause of poor health and consequently one of the most critical public health challenges of the 21st century [1]. Given the major impact of dietary habits on health, it is important to use appropriate dietary assessment tools (DATs) to investigate overall diet quality and food patterns rather than focusing on single nutrients [1,2]. The choice of an appropriate DAT to determine dietary risk factors therefore depends on its ability to assess diet over a time period sufficient to reveal long-term behaviors and patterns [3]. Both diet history and FFQ can measure mid- to long term habitual food intake, in contrast to more short-term measures such as 24-hour recall (24HR) or dietary record (DR), both of which focus only on recent dietary intake [3]. Compared to diet history, FFQs are easier to administer and require less resources, making them the instrument of choice for the measure of long-term dietary intake in large epidemiologic studies [3]. Nevertheless, FFQs are prone to many systematic errors that may affect their validity and reproducibility. Some examples of sources of bias include 1) omission of commonly eaten foods if the list of food items is small or not-culturally specific, leading to an underestimation of absolute levels of consumption of nutrients and food groups [4], 2) inclusion of a large number of food items in the food list (i.e. over 100 food items) can lead to an overestimation of absolute levels of consumption [3], 3) unintentional dietary underreporting due to difficulties in recalling food intake and/or correctly estimating food portions[5], 4) intentional dietary under-reporting due to misreporting of the overall food intake or intake of high calories foods [3], and 5) choosing the time interval for the administration of the FFQ when assessing its reproducibility, where longer intervals may reduce the reproducibility of the FFQ if there are important variations in food intake. Consequently, precautions to reduce measurement errors must be taken at each step, including the design, analysis, and interpretation of the study results [4,6]. Validation of FFQs prior to their use in dietary assessment studies is therefore required to ensure their accuracy. Typically, the validation of FFQs relies upon their comparison with other self-reported DATs as reference methods, such as repeated 24HRs or DRs because of their higher precision in estimation of intake [3]. The emergence of online FFQs has aided in resolving a number of errors encountered with print FFQs, such as missing data, skipped questions or data entry errors [7]. They are also preferred to print-FFQs by users as reported by numerous usability studies [8–10].

The United Arab Emirates (UAE) is witnessing an alarming increase in diet-related NCDs, with 17.3% of the adult Emirati population living with diabetes [11] and CVD responsible for 77% of all deaths [12]. However, data on dietary intake specific to the UAE is scarce mainly due to lack of country-specific DATs that can assess the diet of Emirati nationals. Only one FFQ was developed 16 years ago for the assessment of usual dietary intake of both the UAE and Kuwait, therefore not specifically for the UAE [13]. Moreover, this FFQ was validated in Kuwait but not in the UAE [14]. To remedy the lack of current and specific DAT in the UAE, we have developed a web-based FFQ, the Adult Emirati food frequency questionnaire (AE-FFQ), to provide researchers in the UAE with a reliable and cost-effective tool that can be used in epidemiological studies to assess nutrition related to NCDs in adult Emiratis. As a novel FFQ, the AE-FFQ must first be validated in the population of interest to ensure its adequacy for use as a dietary assessment tool. Therefore, the objective of this study was to assess the relative validity of the AE-FFQ against three 24-hour recalls in a sample of Emirati adults.

## Materials and methods

### Study design

**Fig 1:**
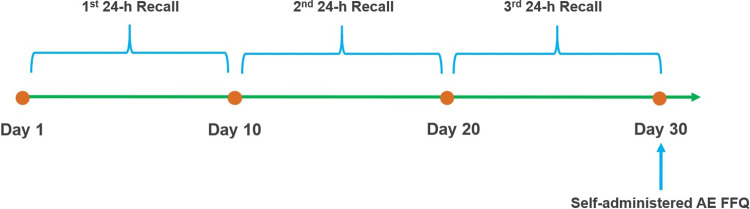
Design of the validation study of the AE-FFQ against three 24HRs among 60 adult Emiratis. 24HR = 24-hour dietary recall; AE-FFQ = Adult Emirati food frequency questionnaire.

This validation study was based on a cross-sectional study design and took place over a one-month period. The majority of the participants were working individuals and were not willing to commit to providing weighted food records due to time constraints. Thus, after providing their informed consent, participants were invited to take part in 3 non-consecutive 24HRs over a period of a month, followed by the AE-FFQ at the end of the one-month period (Fig 1). The study was conducted according to the guidelines of the Declaration of Helsinki. All procedures involving human subjects were approved by the UAEU Human Medical Research Ethics Committee, Al Ain, UAE, where the study was conducted (Ref. No. SS/fa/17-06).

### Participants and recruitment

Recruitment was conducted at the United Arab Emirates University (UAEU) and two other government offices in the city of Al Ain, because Emirati nationals usually work as government employees. They were solicited face to face and 83 adult Emiratis between the ages of 18 and 60 years agreed to take part in the study. Participants were included if they reported consuming a typical Emirati diet, which is an omnivore diet, made of a combination of specific local and cultural foods alongside other international cuisines [15]. With the intention of measuring habitual food and beverage intake for the general Emirati population, exclusion criteria included: Being on a non-omnivore diet (i.e., flexitarian, vegetarian, vegan), or on a special diet for weight loss or for any medical reason, not keeping a consistent weight during the past 3 months and pregnancy or breastfeeding. Demographic information, including age, sex, weight, height, education alongside verbal and written consent was collected in person during the first 24HR. Weight was measured during the first 24HR interview, while height was self-reported.

Of the 83 participants, 81 were eligible for recruitment in this study, 75 were administered both the three 24HRs and the online AE-FFQ, and 60 (72%) were included in the final dataset after excluding misreporters, as described in the flow diagram of the study (S1 Fig).

### Administration of 24-hour recalls

The reference instrument consisted of three unannounced and face-to-face 24HR interviews conducted by the principal investigator and one trained interviewer. One recall was scheduled randomly every 10 days for each participant during the 30-day study period. To account for intra-individual variability in food intake, one of the three interviews captured the intake of a weekend day. During the recall interviews, effort was made to obtain as much information as possible about the foods and beverages consumed by the participants over the previous 24 hours (reflecting the time from midnight to midnight of the previous day), to aid in precise pairing with a the identical or a similar food in a food composition database (FCDB) [16]. To minimize interviewer bias, interviews were conducted using a structured approach inspired by the five steps of the multiple-pass method: (1) quick list, (2) forgotten foods list, (3) time and occasion, (4) detail and review, and (5) final review probe [17]. During the detailed cycle of the multiple-pass method, the digital food images obtained for use in the AE-FFQ were presented to the participants in a PowerPoint slideshow on a laptop to assist them with reporting portion sizes of the foods they consumed.

### Administration of the AE FFQ

The AE-FFQ is a quantitative, self-administered, web-based DAT optimized for use on a laptop in Arabic. Its format was based upon the validated online ‘food4me’ FFQ [18]. Food consumption data from the 2009–2010 national nutrition survey was used to build a culture-specific and comprehensive food list [19]. At the end of the 1-month study period, the participants were sent an email invitation to access the AE-FFQ with a link provided (https://foodfrequencymiddleeast.com) alongside a unique login and password. On accessing “Section 1” of the AE-FFQ, participants were inquired about their usual food intake over the previous month. This section comprised 135 food line-items, divided into 12 groups (Table 1): (1) dairy foods; (2) composite dishes; (3) proteins, including vegetarian and animal sources; (4) vegetables; (5) cereals (including pasta and other cereals), rice, and starches; (6) sandwiches and baked snacks; (7) breads and savory biscuits; (8) spreads on breads, vegetables, or salads, excluding use in cooking; (9) soups; (10) fruits and dried fruits; (11) beverages; and (12) sweets and other snacks (detail of the food list Table 1). Each food line presented a range of seven portion size options made by the combination of three food images of increasing size alongside four additional radio buttons indicating a portion size that was larger/smaller than those shown in the food images [20]. Frequencies of intake options were “Never or less than once per month”, “1–2 times per month”, “3 times per month”, “once per week”, “2 times per week”, “3 to 4 times per week”,” 5 to 6 times per week”, “once per day”, “2 times per day” and “3 times per day”. Four items habitually consumed on a daily basis (water, sugar added to beverages, table salt added to foods, and evaporated milk added to hot beverages) were grouped in a separate section called “Foods Consumed Daily” and presented the same portion size range as in “Section 1” and 7 frequencies (ranging from less than once daily to 6 times daily), resulting in a total of 139 quantitative questions in the AE-FFQ. The other sections of the AE-FFQ on food preference, eating habits, fats used for cooking and dietary supplement consumption were not included in this validation study.

**Table 1:** List of all the 139 foods within the 13 food groups in the AE-FFQ.

Food groups	Dietary items within each group
**Dairy foods**	Cow Milk: full-fat, low fat, or skimmed (1), Yogurt: full-fat, low fat, or skimmed (2), Laban Up (Buttermilk based drink) (3), Butter milk (4), Sandwich cheese sliced; Cheddar cheese; full-fat or low fat (5), Feta Cheese; Halloumi cheese; full-fat or low fat (6).
**Composite dishes**	Mutabbel (Middle-Eastern eggplant-based dip) (7), Tabbouleh (Middle-Eastern parsley-based salad) (8), Hummus (chickpeas dip) (9), Stuffed grape leaves (10), Stuffed Marrow (11), Stuffed Cabbage (12), Harees (Middle-Eastern Pudding-like crushed wheat kernels) (meat or chicken) (13), Margooga; Thareed (Soaked-bread based mixed dishes) (14), Salona (Traditional stew) (15), Cooked mixed rice (16), Macaroni with Bechamel (17).
**Proteins, including vegetarian and animal sources of proteins and processed meats**	Baked beans (18), Eggs (19), Lamb/beef, cooked (20), Lamb/ beef, grilled (21), Camel meat, cooked (22), Chicken, cooked (23), Chicken, grilled (24), Chicken, fried (25), Organ meat e.g. Liver (26), Fried fish (27), White fish, cooked (28), Grilled fish (29), Oily fish (30); Sea food; grilled or fried or cooked (31), Malleh Fish (Salted fish) (32), Mortadella; Salami; Luncheon meat (33), Hot dog (34).
**Vegetables**	Carrots (35), Potato (36), Broccoli; cauliflower (37), Cabbage (38), Cucumber (39), Green peas (40), Green beans (41), marrow (42), Lettuce (43), Green vegetables (44), Tomato, raw (45), Onion, raw (46), Green pepper (47), Pumpkin, sweet potato (48), Eggplant (49), Okra (50), Sweet corn (51), mixed vegetables (52).
**Cereals (pasta and other cereals), rice, and starches)**	Non-sugar-coated cereals (53), Sugar coated cereals (54), Wholegrain cereals, White rice (55), Pasta (56), French fries (57), Oats (58), Pizza (59).
**Sandwiches and baked snacks**	Falafel (Chickpeas-based deep-fried patties) (60), Sambosa (Deep fried pastry) (61), Pakora (Vegetable-based fried snack) (62), Arayes (Meat stuffed pita bread) (63), Fatayer (Stuffed or topped pie) (64), Shawarma (Middle-Eastern sandwich made with rotisserie meat) (65), Hamburger (meat or chicken) (66).
**Breads and savory biscuits**	White bread; slice (67), Brown Bread; slice (68), Rgag Bread (69), Arabic Bread (70), Chebab bread (Emirati pancake) (71), Samoon Bread (Hot Dog bread or bread roll) (72), Chapati (unleavened flat brad) (73), Fried bread, e.g. Paratha or Puri (74), Crackers and Salted biscuits (75).
**Spreads on breads, on vegetables, or on salads.**	Labneh (Cheese spread) (76), Ghee (clarified butter) (77), Butter (78), mayonnaise; salad cream (79), Jam (80), Honey (82), Chocolate spread (83), Dates syrup (84), Lemon juice (85), Ketchup or tomato sauce (86), Hot chili sauce (87), Pickles or chutney (88), Olives (89).
**Soups**	Soup of vegetables only (90), Soup with Meat or chicken (91), Soup with legumes (92), Instant dehydrated soup (93), Instant noodles soup (94).
**Fruits & Dried Fruits**	Apple; pear (95), Banana (96), Orange; tangerine (97), Strawberries; blueberries (98), Pineapple (99), Pomegranate (100), Grapes (101), Kiwi (102), Plum or peaches (103), Mango (104), Watermelon; melon (105), Fruit salad (106), Dates (107).
**Beverages**	Soft drinks, sweetened (108), Soft drinks, unsweetened (109), Fruit cocktail, sweetened (110), Fruit juice, unsweetened (111), Energy drinks (112), Milk shakes; smoothies (113), Fruit-Flavored drink (114).
**Sweets and desserts**	Um Ali (Egyptian bread pudding) (115), Kunafah (Middle-Eastern pastry filled with cheese or cream) (116), Balaleet (Emirati Sweet Vermicelli and Eggs) (117), Lgeimat (Emirati Fritters) (118), Qurs (Emirati 0fried sweetened bread) (119), Omani Halwa (Omani Sweet jelly dessert) (120), Rahash, Halwa (Middle-Eastern Tahini-based sweet) (121), Baklava (Middle-Eastern Phyllo pastry with spiced walnuts) (122), Maamoul (Middle-Eastern date filled cookies) (123), Pudding (124), Biscuits or cookies (125), Cakes (126), donuts (127), croissants (128), Cakes with cream (129), Chocolate bars (130), Ice cream (131), Potato chips (132), Salted sunflower seeds (133), mixed nuts (134), Pop-corn (135)
**Foods consumed daily**	Water (136), sugar added to beverages (137), Evaporated milk (138), salt added at the table (139).

### Analysis of nutrients and food groups intake

The nutrition analysis software chosen for processing the food consumption data was Nutritionist Pro (version 7.5.0, Axxya Systems, Stafford, TX, USA), as it contains a larger choice of international FCDBs compared to other commercial nutrition analysis programs (e.g., ESHA) [21]. The following procedure was used for matching all 532 foods that were reported in the three 24HRs and the food list of the AE-FFQ. Since the UAE does not currently have food composition tables (FCT) and only few traditional dishes have been chemically analyzed [22], we have decided to use different sources of nutrient data to match the foods reported in the three 24HRs and in the food list of the AE-FFQ. The methodology we used for matching the foods reported has been described elsewhere [23]. Briefly, we strived to match the foods reported with the best food match from a FCDB that fulfilled the Food and Agriculture Organization/International Network of Food Data Systems (FAO/INFOODS) guidelines for food matching [24], which requires having an identical or similar food name and edible form as well as a complete list of nutrient values of interest that are expressed in standardized definitions, expressions, units and denominators [24]. The reference nutrient data source used for food matching was the United States Department of Agriculture National Nutrient Database for Standard Reference database (USDA SR DB) because of its high-quality and completeness [25]. When a perfect food match was not possible in the SR DB, a consistent and standardized stepwise approach was implemented to ensure the best possible food match [23]. Based on the above criteria, Table 2 outlines the different FCDBs used, and some examples of the corresponding foods matched.

**Table 2:** Nutrient data sources and their contribution to the matching of the 532 foods reported in the survey.

Nutrient data source	Examples of foods that were best matched in the Nutrient data source
USDA SR DB	• Single ingredient foods (e.g., fruits and vegetables, meat by its different cut types, etc) • Basic multiple-ingredient foods (e.g. sliced bread) • US branded food products (e.g. Pizza hut™) • Generic food products (e.g. ketchup, carbonated drinks)
FNDDS	• Foods from mixed dishes (e.g. chocolate filled crepes, Beef hotdog wiener)
CNF	• Mixed dishes not found in the FNDDS (e.g. squid, flour coated, fried, vegetarian samosa, sushi with fish and vegetables)
UK DB	• Some Western foods with cooking methods not found in the North American DBs (e.g. grilled salmon) • Indian and Middle Eastern mixed dishes (e.g. stuffed grape leaves, Punjabi Puri, Bombay Mix) • UK branded food products (e.g. Digestive biscuits™)
New Zealand DB	• Branded Food products (e.g. Pringles™, Hazelnut spread Nutella™, Ferrero™, Indomie™ Maggie chicken Noodles)
Chemically analyzed Emirati foods	• Emirati breads (e.g. Qurus, Khameer, Chebab, Rgag, Muhalla) • Emirati cheese (Chami cheese) • Emirati desserts (e.g. Balaleet, Lgeimat)
Swedish DB	• Homemade fried Meatballs • Branded food: La Vache qui rit™
French DB	• Roasted/baked tomatoes with skin)
Kuwaiti DB	• Arabic fish dish (Malleh)
Greek DB	• Baklava
Irish DB	• Branded food: Breakfast cereal Alpen™ Wheat Flakes

USDA SR DB = United States Department of Agriculture Standard Reference Database, FNDDS = Food and Nutrient Database for Dietary Studies, CNF = Canadian Nutrient File, UK = United Kingdom, DB = Database.

The use of multiple high-quality international FCDBs [25–28] that contained values for all our components of interest was an adequate approach for the UAE, which imports up to 90% of its food from various countries, particularly the United States and United Kingdom [29]. This methodology was preferred to using a unique FCDB and completing it by borrowing missing component values from other data sources as the latter method is more susceptible to introducing bias due to errors in the calculations required. Food composition that could not be taken from the data sources described above (e.g., Emirati cookies or other pastries) were obtained by recipe calculation, taking into account the appropriate yield and retention factors [30,31]. This process made missing values of nutrient estimates negligible.

Some of the line-items in the AE-FFQ contain more than one food, for example oranges and tangerines (Item 97 in Table 1) were aggregated in a single line as “citrus fruits”. To assign nutrient values to composite line-items containing more than one food, the following procedures were performed: 1) We obtained food consumption data of each food within the composite line from the 2009/2010 UAE dietary intake survey, which assessed the food intake of adult Emirati women [19], 2) Calculated the weight for each food within the line based on its consumption relative to the other foods within the line such as: Relative consumption of food X = (Consumption of X/Total consumption of all foods in the line), 3) Obtained the weighted mean of the composite line by summing the relative weights of the nutrient values of all the foods within the line.

To estimate the daily energy and nutrient intake from the AE-FFQ the following calculation was applied:

Daily nutrient intake in grams = Sum [(daily consumption frequency of a food line item) × (weighted average portion size consumed for that food-line item (in grams) x component value/100 g)]. Daily consumption frequencies were obtained by multiplying the frequencies reported in the AE-FFQ by a specific factor, for example: Never = 0; 1–3/month = 0.07; 1/week = 0.14; 2–4/week = 0.43; 5–6/ week = 0.79; 1/day = 1.0; 3/day = 3 [3].

The AE-FFQ was designed to not allow for skipping questions, thus resolving an important source of error due to missing data. To limit bias due to misreporting of the three 24HRs, we excluded from the final analysis participants for whom the energy intake reported on the three 24HRs was outside of the range of 800 to 3500 kcal for women or 1000 to 4000 kcal for men [24]. Willet notes that intakes of more than 4,000 kcal/day are unlikely even in relatively active men [3]. This method is simple and generally preferred to the Goldberg method (which depends on body weight to estimate energy requirements) because it avoids the selection bias caused by individuals with higher BMIs who tend to report low energy intakes [32].

To enable comparison of food groups’ intake between methods, the 139 food lines in the AE-FFQ were re-classified into 31 food groups. Foods from the 24-hour recall data were then matched to the most appropriate food group. The food groups assessed in the validation study were similar to the ones used in other studies sharing the same objective of validating an FFQ aimed for use in research on dietary risk factors of CVDs [18,20,33]. Most of the food groups had an evidenced potential protective (e.g., vegetables or fruits food groups) or adverse (e.g. sugary beverages, red meat groups) effect on NCDs [1] (S1 Table). When a reported food from the 24HRs or a food from the FFQ’s food list did not fit the exact food group description, it was assigned to the closest group, e.g., “hash browns” were assigned to the ‘French fries’ group. Composite foods in their cooked form were split into their basic ingredients and then assigned to their corresponding food group. All coding was performed by the principal investigator.

### Sample size calculation

Based on Thompson and Byers review [34] that indicated that correlation coefficients between FFQ and a reference instrument for most foods and nutrients were within the range of 0.4 to 0.7, we used power analysis for correlation coefficients to generate the minimum sample size [35]. For a desired minimum correlation coefficient of 0.4 between the AE-FFQ and the three 24HRs (at α = 0.05 and 95% power), the sample size obtained was 59 output of the power analysis in shown in S2 Fig).

### Statistical analysis

Normality tests (Shapiro–Wilk test, Kolmogorov–Smirnoff test, and Q-Q plot) performed at the beginning of the data analysis for all nutrients and food groups of the AE-FFQ and the three 24HRs showed a clear deviation from normality for most variables. Consequently, validity was assessed with nonparametric tests, except for Bland–Altman analysis. Interpretation of validity tests was based on the guidelines outlined by Lombard et al. [36].

Medians, interquartile ranges (IQR), means and standard deviations (SD) were calculated for energy, nutrients and food groups intakes. Wilcoxon signed rank sum test was used to compare differences between the matched measures. Percentages of the difference in mean intake between the two methods were calculated based on this formula ([mean (FFQ–three 24HR)]/[mean (three 24HR) ×100), and a percentage of the mean difference <10% signaled good agreement between the methods based on the Lombard criteria [36]. Agreement between the two methods at the group level was assessed by Bland–Altman analysis. Given that data were not normal, natural log (ln) transformations were performed as recommended by Bland and Altman [37]. All analyses were carried out on energy-adjusted variables obtained by the residual method to provide values that are not confounded by total energy intake (EI) [38]. Visualization of the limits of agreement (LOA) (ln mean difference ± 1.96 SD) between the methods was performed by plotting the difference between the AE-FFQ and the three 24HRs against the mean (ln) of the two methods [37]. A good agreement between the methods was obtained when 95% of the differences fell within the LOA [36]. The regression line of difference, in which the three 24HR was a predictor of AE-FFQ was fitted on the plots to detect and visualize any dependency between the methods [39]. The strength and the direction of the association at the individual level between energy, nutrients, and food groups reported by the two methods was assessed using crude, deattenuated, energy-adjusted, and deattenuated energy adjusted by Spearman correlation coefficients (SCCs). To remedy the random error caused by to day-to-day variation in the three 24HRs, deattenuated SCCs were obtained by multiplying each crude SCC by a deattenuation coefficient obtained using following formula:

√1+[(σw2/σb2)/n]

where σw2 is the within person variance, σb2 is the between-person variance, and n is the number of replicates of the reference instrument.

For this study, n = 3, representing each of the 24HRs [40]. The residual method was used to calculate energy-adjusted and deattenuated energy-adjusted SCCs [38]. Lombard et al. [36]. criteria consider a CC ≥0.50 as good, 0.20–0.49 as acceptable, and <0.20 as poor. Categorical agreement was assessed by using quartile classification of energy-adjusted intake of each nutrient and food group from both methods to estimate the percentage of participants that could be correctly categorized into the same or adjacent (±1) quartiles or misclassified into the extreme (opposite) quartile [41]. Lombard et al. consider an outcome of >50% of participants classified into the same quartile as good versus an outcome of misclassified for <10% of participants in the opposite quartile [32]. All statistical analyses were performed using Python 3.7.7 and SPSS for Windows (version 23, SPSS, Chicago IL, USA). A value of p < 0.05 was considered significant, and all tests were performed two-sided.

### Ethics approval and consent to participate

All participants were informed about their role in the study and they gave verbal and written informed consent before the start of the study. All procedures involving human subjects were approved by the United Arab Emirates University Human Research Ethics Committee, Al Ain, UAE, where the study was conducted (Protocol No. SS/fa/17-06).

## Results

### Sample characteristics

Table 3 depicts the anthropometric and socio-demographic characteristics of the study participants who were included in the analysis compared to those who were eligible but were not included in the final dataset.

**Table 3:** Sociodemographic profile of the validation study participants and non-participants.

	Participants (n = 60)			Non-Participants (n = 21)			
Characteristics	Males n (%)	Females n (%)	Total n (%)	Males (%)	Females (%)	Total n (%)	p Value*
**Age in years (Mean ± SD)**	33.13 ± 10.12	32.69 ± 7.41	32.87 ± 9.13	33.25 ±7.17	32.23 ± 8.99	32.62 ±8.29	0.02
**Age groups (Years)**							
21–30	13 (54.2)	17 (47.2)	30 (50.0)	4 (50.0)	6 (46.2)	10 (47.6)	
31–40	5 (20.8)	13 (36.1)	18 (30.0)	3 (37.5)	5 (38.46)	8 (38.1)	
41–50	3 (12.5)	6 (16.7)	9 (15.0)	0 (0)	1 (7.7)	1 (4.8)	
51–60	3 (12.5)	0 (0)	3 (5.0)	1 (12.5)	1(7.7)	2 (9.5)	
**Education**							0.855
Graduate	6 (25.0)	2 (5.6)	8 (13.3)	1 (12.5)	1 (7.7)	2 (9.5)	
Undergraduate	13 (54.2)	29 (80.6)	42 (70.0)	4 (50.0)	8 (61.5)	12 (57.1)	
High School	3 (12.5)	5 (13.9)	8 (13.3)	2 (25)	3 (23.1)	5 (23.8)	
Less than high school	2 (8.3)	0 (0)	2 (3.33)	1 (12.5)	1 (7.7)	2 (9.5)	
**BMI (Kg/Meter** ^**2**^ **) (Mean SD)**	26.66 ± (5.60)	25.19 ± (4.28)	25.78 ± (4.86)	25.41± (3.10)	25.85 ± (3.77)	25.69 ± (3.46)	0.370
**BMI (Kg/Meter** ^**2**^ **), in categories**							
18.5–24.9 (Normal)	11 (45.8)	22 (61.1)	33 (55.0)	5 (62.5)	6 (46.15)	11 (52.4)	
25–29.9 (Overweight)	6 (25.0)	9 (25.0)	15 (25.0)	2 (25)	4 (30.8)	6 (28.6)	
30 or more (Obese)	7 (29.2)	5 (13.9)	12 (20.0)	1 (12.5)	3 (23.1)	4 (19.0)	
**Total (%)**	24 (40.0)	36 (60.0)	60 (100.0)	8 (38.1)	13 (61.9)	21 (100.0)	

BMI = body mass index (Kg/m^2^); SD = standard deviation. The *p* value is based on chi-square test for categorical variables and independent t-test for continuous variables.

### Measure of validity

Tables 4 and 5 depict the mean and standard deviation (SD), Spearman’s correlation coefficients and the agreement between the methods for nutrients and food groups respectively.

**Table 4:** Mean daily nutrient intakes, Spearman correlation coefficients and % agreement from the average of three 24HRs and the AE-FFQ (n = 60).

Nutrients Intake (Units/day)	AE-FFQ	Mean three 24HR	Percentage of mean difference	*p* value	Spearman Correlation			% agreement
	Mean ± SD	Mean ± SD			Crude	Deattenuated	Energy-Adjusted & Deattenuated	
Energy (kcal)	2948.3 ± 1346.6	2169.3 ± 522.3	36%	<0.001	0.54*	0.59*	--	69.48
Protein (g)	121.5 ± 72.7	84.5 ± 29.2	44%	<0.001	0.52*	0.57*	0.39*	76.27
Total carbohydrate (g)	383.7 ± 173.2	282.2 ± 70.0	36%	<0.001	0.42*	0.46*	0.32	64.39
Total Sugar (g)	119.7 ± 60.7	90.5 ± 33.4	35%	<0.001	0.60*	0.65*	0.60*	62.7
Dietary fiber (g)	29.5 ± 14.6	18.4 ± 6.6	32%	<0.001	0.50*	0.61*	0.62*	76.26
Total fat (g)	110.7 ± 55.4	82.4 ± 24.7	40%	<0.001	0.42*	0.46*	0.32	74.58
SFA (g)	39.2 ± 20.5	28.1 ± 8.8	24%	<0.001	0.33*	0.37*	0.26	72.87
MUFA (g)	37.9 ± 19.8	30.5 ± 10.1	37%	0.013	0.38*	0.41*	0.38*	71.18
PUFA (g)	25.0 ± 14.6	18.3 ± 6.7	61%	<0.001	0.46*	0.50*	0.44*	69.48
Cholesterol (mg)	419.6 ± 282.4	284.1 ± 119.7	48%	<0.001	0.48*	0.53*	0.54*	61.02
Sodium (mg)	4548.7 ± 2046.2	3103.1 ± 1069.4	47%	<0.001	0.53*	0.62*	0.57*	69.48
Calcium (mg)	1057.4 ± 524.1	707.5± 205.0	49%	<0.001	0.42*	0.50*	0.44*	62.71
Iron (mg)	19.8 ± 9.7	17.8 ± 18.2	11%	0.005	0.13	0.13	0.06	59.31
Vitamin A (mcg)	1072.0 ± 543.9	781.9 ± 569.1	37%	<0.001	0.11	0.12	0.10	64.92
Vitamin B12 (mcg)	8.3 ± 5.9	4.6 ± 4.5	81%	<0.001	0.42	0.47	0.43	71.19
Vitamin C (mg)	251.8 ± 151.6	144.1 ± 120.7	75%	<0.001	0.42*	0.48*	0.47*	69.3
Vitamin D (mcg)	6.9 ± 5.4	4.9 ± 4.2	40%	0.034	0.19	0.20	0.19	64.39
Vitamin E (mg)	12.6 ± 8.3	13.4 ± 31.8	-6%	0.006	0.49	0.49	0.09	69.49
Thiamine (mg)	4.2 ± 3.0	3.2 ± 2.0	31%	0.016	0.26	0.27	0.26	55.92
Riboflavine (mg)	5.1 ± 6.4	2.9 ± 5.2	73%	<0.001	0.32	0.33	0.18	77.95
Pyridoxine (mg)	3.5 ± 1.9	2.5 ± 1.1	41%	<0.001	0.40*	0.44*	0.45*	64.41
Folate (mcg)	434.0 ± 266.3	255.6 ± 132.1	70%	<0.001	0.40*	0.47*	0.49*	33.9
Median	--	--	--	--	0.42*	0.47*	0.39*	69.39

The *p* value of the mean difference is based on the Wilcoxon signed-rank test.

*p < 0.05; 24HR = 24-hour dietary recall; AE-FFQ = Adult Emirati food frequency questionnaire; MUFA = monounsaturated fatty acids; PUFA = polyunsaturated fatty acids; SD = standard deviation; SFA = saturated fatty acids.

**Table 5:** Mean daily food groups intakes, Spearman correlation coefficients and % agreement from the average of three 24HRs and the AE-FFQ (n = 60).

Food Groups (in grams)	AE-FFQ	Mean three 24HR	Percentage of mean difference	p value	Spearman Correlation			% agreement
	Mean ± SD	Mean ± SD			Crude	Deattenuated	Energy-Adjusted & Deattenuated	
Dairy drinks	140.3 ± 204.0	104.4 ±111.8	34%	0.161	0.47*	0.48*	0.42*	76.66
Cheeses (Hard and spreadable)	38.4 ± 35.3	20.9 ± 16.8	37%	0.001	0.34	0.37	0.37	70
Yoghurts	61.4 ± 74.5	59.5 ± 74.7	3%	0.705	0.46*	0.46*	0.46*	81.66
Rice dishes	335.0 ± 319.1	210.7 ± 146.1	59%	<0.001	0.67*	0.71*	0.62*	66.66
Pasta & other cereals dishes	45.7 ± 48.8	29.2 ± 47.8	57%	<0.001	0.54*	0.56*	0.47*	81.66
White breads	119.6 ± 87.5	93.9 ± 59.8	27%	0.035	0.22	0.23	0.22	68.33
Wholegrain breads	11.5 ± 23.9	4.8 ± 12.6	143%	0.018	0.44*	0.45*	0.35*	63.33
Legumes	35.1 ± 44.4	17.8 ± 26.5	97%	<0.001	0.44*	0.46*	0.41*	61.66
Eggs	35.6 ± 36.8	24.1 ± 34.0	48%	0.001	0.68*	0.70*	0.65*	81.66
Red meat (not including processed meat, sausages)	40.7 ± 36.8	30.0 ± 44.9	36%	0.058	0.45*	0.46*	0.41*	68.33
Meat products (Hot dogs, sausages)	49.3 ± 63.7	48.3 ± 59.8	2%	0.906	0.52	0.52	0.43	83.33
Chicken dishes	68.8± 72.2	64.9 ± 54.2	6%	0.985	0.41*	0.41*	0.31	66.6
Fish & Seafood	78.3 ± 112.5	25.2 ±40.9	210%	<0.001	0.50*	0.54*	0.49*	61.66
Total vegetables	250.8 ± 238.0	118.8 ±106.7	111%	<0.001	0.57*	0.64*	0.56*	61.66
Greens	25.7 ± 29.7	15.9 ±14.9	62%	0.003	0.50*	0.53*	0.47*	61.66
Cruciferous vegetables	16.5 ± 27.8	5.6 ± 13.3	196%	0.001	0.31	0.33	−0.02	75
Red or yellow vegetables	68.9 ± 66.7	41.8 ± 32.2	65%	0.002	0.46*	0.49*	0.42*	63.32
Potatoes	21.9 ± 34.3	11.7 ± 22.5	87%	0.035	0.36	0.37	0.32	61.66
Other vegetables	117.8 ± 138.4	43.9 ± 58.3	169%	<0.001	0.40*	0.45*	0.42	59.99
Savory snacks (Fatayer, falafel, croissants)	46.5 ± 60.8	46.0 ± 36.3	1%	0.612	0.35*	0.35*	0.31	81.66
Fruits	224.4 ± 185.5	98.7 ± 111.9	127%	<0.001	0.42*	0.49*	0.36*	64.99
Dried fruits	20.3 ± 25.7	14.5 ± 20.0	40%	0.015	0.62*	0.63*	0.60*	76.67
Soft drinks	58.9 ± 151.3	74.6 ± 117.5	-21%	0.065	0.54*	0.54*	0.60*	86.66
Diet soft drinks	7.1 ± 32.9	4.3 ± 27.0	64%	0.600	0.30*	0.30*	0.43*	55
Fruit juices, including smoothies	118.1 ± 156.6	138.3± 158.7	-15%	0.181	0.35*	0.35*	0.34*	66.66
Sugar, syrups, jams, honey	16.2 ± 16.5	9.3 ± 7.7	75%	<0.001	0.50*	0.54*	0.47*	66.66
French fries	26.5 ± 35.2	26.7 ±32.4	-1%	0.821	0.48*	0.48*	0.39*	74.99
Sweet snacks (Biscuits, Arabic sweets)	38.3 ± 34.3	37.9 ± 38.5	1%	0.960	0.44*	0.44*	0.38*	73.33
Sweets, candies, and Chocolates	12.1 ± 23.9	11.8 ± 16.9	2%	0.766	0.31*	0.31*	0.21*	64.99
Chips	7.6 ±13.3	5.0 ± 8.4	53%	0.242	0.46*	0.47*	0.27*	69.99
Nuts and seeds	14.6 ± 23.8	11.0 ± 17.8	33%	0.462	0.44*	0.45*	0.35*	64.99
Median	--	--	--	--	0.45*	0.46*	0.41*	66.66

The *p* value of the mean difference is based on the Wilcoxon signed-rank test.

*p < 0.05; 24HR = 24-hour dietary recall; AE-FFQ = Adult Emirati food frequency questionnaire.

In general, the AE-FFQ significantly overestimated (*p* < 0.05) energy and most nutrients compared to the three 24HRs, with the exception of vitamin E. Of the 31 food groups assessed, 17 were significantly overestimated (*p* < 0.05). Discrepancies between the methods ranged from -6% (vitamin E) to 81% (vitamin B12) for nutrients and from 210% (fish and seafood group) to -21% (soft drinks) (*p* < 0.05). The crude SCC showed a median value of 0.42 for nutrients. Accounting for the day-to-day intake variations resulted in a deattenuated median SCC of 0.47. Energy-adjustment reduced the correlations of most nutrients, and energy-adjusted and deattenuated SCC values ranged from 0.06 (iron) to 0.62 (fiber), with a 0.39 median value. For food groups, the crude correlations showed a 0.45 median value. As observed with nutrients, the median correlation increased slightly (0.46) after deattenuation and decreased to a 0.41 median after energy adjustment, with energy-adjusted and deattenuated correlations ranging from –0.01 for cruciferous vegetables to 0.64 for eggs.

The percentage of participants classified into the same or adjacent quartile ranged from 34% (vitamin B12) to 78% (pyridoxine) (median 69%) for nutrients and from 55% for diet soft drinks to 87% for soft drinks (median 67%) for food groups. The percentage of participants classified in opposite quartiles was within the guidelines for most nutrients but exceeded 20% for cruciferous vegetables and diet soft drinks.

**Fig 2:**
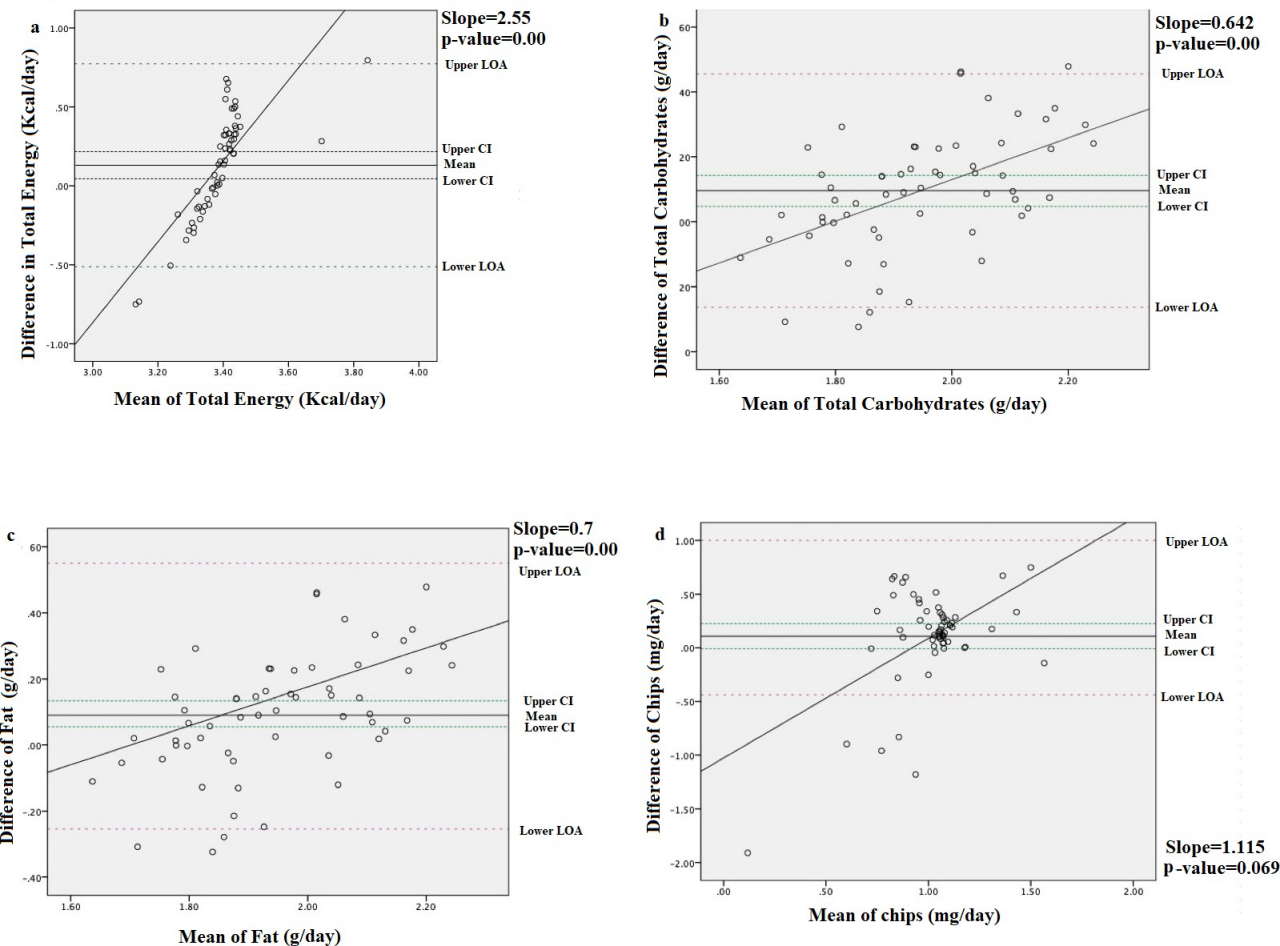
Bland–Altman plots for energy intake, selected energy-adjusted nutrient and food group intakes showing the varying levels of agreement obtained between mean (ln) and differences in intakes measured by the AE-FFQ and the three 24HRs: (a) energy intake (Kcal/day); (b) total carbohydrate, (c) total fat(g/day); (d) chips 24HR = 24-hour dietary recall; AE-FFQ = Adult Emirati food frequency questionnaire; ln = natural log.

**Fig 3:**
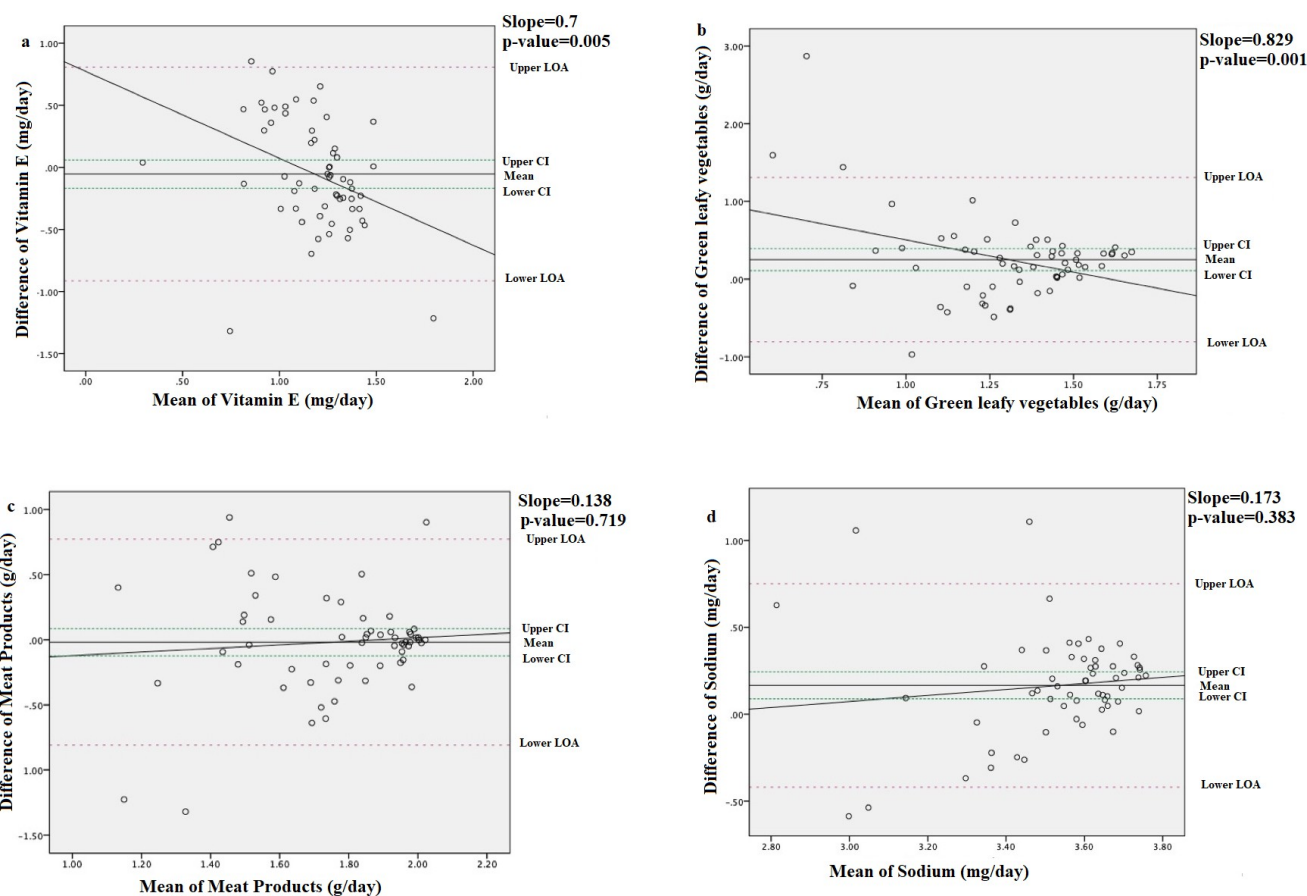
Bland–Altman plots for selected energy-adjusted nutrient and food group intakes showing the varying levels of agreement obtained between mean (ln) and differences in intakes measured by the AE-FFQ and the three 24HRs: (a) vitamin E (mg/day); (b) green leafy vegetables (g/day); (c) meat products(g/day); (d) sodium (mg/day). 24HR = 24-hour dietary recall; AE-FFQ = Adult Emirati food frequency questionnaire; ln = natural log.

Bland-Altman analysis revealed that most mean differences were positive and significant (*p* > 0.05), for both nutrients and food groups, confirming the overestimation of intake by the AE-FFQ (Figs 2 and 3). There was proportional bias for most nutrients and food groups. As the mean intake increased, the agreement between the methods decreased for EI (Fig 2A), total carbohydrate (Fig 2B), total fat (Fig 2C), and the “chips” food group (Fig 2D). Conversely, the agreement between the methods increased as the mean intake increased for vitamin E (Fig 3A) and the “green leafy vegetables” food group (Fig 3B). A flat line was observed for the food groups “meat products” (Fig 3C) and the nutrient sodium (Fig 3D) indicating that the difference between the methods did not vary with true intake.

## Discussion

This study describes the validation of the first Web-based FFQ developed specifically for the adult Emirati population. Overall, results showed an acceptable validity of the AE-FFQ when compared to the three 24HRs for the population in the City of Al Ain, UAE.

At the group level, a low to moderate agreement between the methods was obtained because most nutrients and food groups showed a percentage of mean differences larger than the 10% threshold that determines good agreement between the methods [36]. A higher percentage of mean differences in the FFQ was also found in a study from Lebanon [42]. The AE-FFQ significantly overestimated energy (mean difference + 779 Kcal/day) and most nutrients compared to the 3-d 24HRs. The overestimation of EI is a tendency that is often expected in comprehensive FFQs, more specifically when the number of food items exceeds 100 [36], as reported in FFQ validation studies from neighboring countries [34,37–40] and in Web-based FFQs [13,41,42]. Tayyem et al. [34] and Dehghan et al. [35] also reported high EI discrepancies (+1,011.2 Kcal/day and 506 Kcal/day respectively). Tayyem et al. [34] argued that the EI overestimation may have been due to the long list of food items, while Dehghan postulated that social desirability and misunderstanding of frequencies and portion sizes may have been the reason for the overestimation of EI [35]. In the AE-FFQ, both the long list of food items and the choice of extra-large portion sizes by some male participants may have caused the observed overestimation of EI. However, other Web-based FFQs reported underestimation [8,43,44] or no difference in estimation of energy and nutrient intake [45]. The AE-FFQ also overestimated the amount of nutrients consumed. The large percentage difference observed with vitamin B12 (+81%) may have been a reflection of the fact that organ meat, which is the highest source of vitamin B12 in the AE-FFQ, is rarely consumed in this population. A similar explanation can be given for the overestimation of the reporting of fish (+210%) and brown bread (+143%). Indeed, Dehghan et al. [13] and Musaigher et al. [15] reported that meat and chicken, but not fish, are the most predominant sources of the animal proteins in the Emirati diet. This trend of overestimation of less frequently consumed foods has been reported in other Web-based FFQs [45,46]. Likewise, foods that showed the highest agreement between the methods were those that are more frequently consumed: sweet and savory snacks, French fries, fruit juices, and soft drinks. Earlier studies have reported the popularity of snacks and fruit juices in the Emirati population [19]. Overreporting of fruits and vegetables by the AE-FFQ is another bias commonly found in validation studies of comprehensive FFQs [5]. The long list of fruits and vegetables may also explain the overestimation observed with fiber (+61%), vitamin A (+37%) and vitamin C (+75%)—all markers of high fruits and vegetable intake [47]. A similar positive association is also reported elsewhere [48].

The strength and direction of the association between the AE-FFQ and the average 24HRs at the individual level for energy, nutrients, and food groups was measured by SCC. Based on Lombard’s interpretation criteria, both crude and deattenuated correlations showed acceptable to good validity for energy, 17 of the 21 nutrients, and all the 31 food groups. When assessed against other published criteria, acceptable validity was maintained for only 15 nutrients and 22 food groups based on the threshold of 0.4 reported by Cade et al [5], and only 4 nutrients and 9 food groups were considered desirable based on the threshold of 0.5 described by Masson et al. [49]. However, after deattenuation, energy, 7 nutrients and 10 food groups presented a good level of association because they were >0.5 [36]. When comparing the range of deattenuated unadjusted correlations obtained for nutrients and food groups with validation studies of FFQs that used 24HRs as their reference instrument, the range of 0.12–0.65 obtained for the nutrients in this study were comparable with those in previous Web-based FFQ validation studies (range, 0.14–0.78 [8,50]) and in FFQs from other Arabic or neighboring countries (range, 0.02–0.73 [14,51,52]). For food groups in the present study, the range 0.22–0.68 was similar to those in other Web-based FFQ validation studies (range, 0.11–0.73 [18,53,54]). Foods with the highest correlations were foods that were consumed almost daily in the Emirati diet (eggs, rice, and dates). Similarly, food groups with the lowest correlations (cruciferous vegetables and diet soda drinks) were not frequently reported in the reference instrument. The correlations of energy, nutrients, and food groups obtained may have been inflated because of the use of the 24HR as the reference instrument, which shares memory bias as a potential source of error [3]. However, despite both methods relying on memory, FFQ investigates long-term memory, whereas 24HR assesses short-term memory [3]. Other errors that may have inflated the correlation results are the use of the same food images to depict the portion size options and the use of the same nutrient data source for both the instruments compared in this study.

Given the possibility of correlated errors between the two instruments, use of energy-adjusted nutrients and food groups was performed [38]. Adjusting for EI is also required to account for confounding effects in studies assessing diet–disease relationships due to energy [30]. Energy-adjustment decreased SCC for almost all nutrients and food groups. Correlations were less than acceptable (<0.2) for 5 nutrients, with iron, vitamin E, and riboflavin correlations showing the largest decrease. For the food groups, an acceptable level of validation was maintained for all groups (>0.2), except for the cruciferous vegetables group (–0.02). A decrease in SCC following energy-adjustment usually occurs when the variability of intake is caused by systematic errors of under or overestimation rather than a high EI of the participants [55]. Previous validation studies of Web-based FFQs [8], and validation studies of FFQs from Arabic or neighboring countries [51,56,57] have also reported that energy-adjusted estimates decreased after energy-adjustment.

Bland–Altman analysis generally showed a fair agreement; however, the AE-FFQ underestimated or overestimated intake for energy and most nutrients and food groups. The AE-FFQ did not perform well for assessing higher intake for most food groups and nutrients, especially EI. This finding may be attributed to the 24HR not being an appropriate reference method because it is not considered the gold standard of reference instruments in validation studies [3]. This finding also suggests that the AE-FFQ is not suitable for assessing absolute intake in the adult Emirati population. However, it can be used to rank individuals based on their nutrient and food group intake, as evidenced by the results of the cross-classification analysis wherein a fairly acceptable agreement was observed with an average of 67% and 69% of the participants correctly classified into the same or adjacent quartile of adjusted intakes for nutrients and food groups respectively, whereas only 8% and 10% participants were classified in opposite quartiles for nutrients and food groups respectively. Other studies [10,14,43,51] have also reported good agreement at the group level wherein nutrients were correctly classified into quartiles, although the agreement in assessing absolute intake was poor.

This study has a number of limitations that should be taken into consideration when interpreting the results. One such limitation is that we were not able to conduct the reproducibility of the AE-FFQ. We planned to conduct the reproducibility a month after the first administration of the AE-FFQ. However, because of delays experienced in the testing of the tool, the month after the first administration of the AE-FFQ coincided with the Islamic month of Ramadan, a period that involves drastic changes in the dietary habits. When attempting to administer the second AE-FFQ a month after the end of Ramadan, the number of active participants left from the initial sample did not reach the minimum required to carry out the reproducibility study with sufficient precision.

Another limitation of this study is that it was conducted on a convenience sample of volunteers in the city of Al Ain, UAE, with most participants being educated, young, and female; therefore, this study population lacks generalizability to the Emirati population more broadly. Since the AE-FFQ is a web-based FFQ, it is not advisable for use in people with low literacy skills or individuals who may not be confident using a computer. In light of these limitations, we recommend that future studies assess the validity and reproducibility of the AE-FFQ in a representative sample of Emirati adults. Although the use of three replicates of the 24HR as the reference instrument in this study is supported by many studies [5,58,59], a larger number of replicates may have helped improve the validity of the AE-FFQ, given that micronutrients showed a consistently lower validity across all statistical tests compared with macronutrients. However, more replicates may also have increased the burden on the participants and may have induced a higher attrition rate. Finally, the use of recovery and/or concentration biomarkers, which have uncorrelated errors may have added valuable information about the validity of the AE-FFQ [3]. Some of the strengths of this study lie in the web-based format of the AE-FFQ, which ensured a fully automated and immediate data output after completion of the AE-FFQ, with no double data entry, and no requirement for data cleaning, thus making the AE-FFQ, to our knowledge, one of the few fully automated self-administered web-based FFQ in the Arab world. The tool did not take more than 30 minutes to complete and was easy to use by the educated participants. Moreover, the tool included a wide range of food photographs to help with estimation of intake because it has previously been shown that the use of a large number of food photographs improves the ability of an individual to more accurately report dietary intakes [60]. Finally, the use of nonparametric methods (Spearman correlation coefficient, Wilcoxon signed-rank test) which are more robust than parametric tests when deviations from normality exist and the small sample size may have contributed to the observed associations, which were fair and statistically significant overall [36].

## Conclusions

The newly developed AE-FFQ showed an acceptable level of validity for most nutrients and food groups. It can be a useful tool for ranking food and nutrient intakes of Emirati adults (aged 18 to 50) in epidemiological studies and thus promoting research on diet-disease relationship in the UAE, while being cautious of the risk of overestimation of intake in the interpretation of the results. These findings need to be confirmed in a more representative sample of adult Emiratis.

## Supporting information

S1 Fig Flow of participants through the validation study. (PDF)

S2 Fig Output of power analysis to determine sample size in G*Power. (PNG)

S1 Table Categorization of the 31 food groups used for comparing food group intakes of the AE-FFQ by the three 24HRs. (PDF)

S1 Raw data (XLS)

## Abbreviations

24HR: 24-h dietary recall
AE-FFQ: Adult Emirati food frequency questionnaire
BMI: Body Mass Index
ICC: Intra-class correlation coefficients
NCDs: Non communicable diseases
SD: Standard deviation
CNF: Canadian Nutrient File
CVDs: Cardiovascular diseases
DALY: Disability-adjusted life-years
DAT: Dietary Assessment Tool
DB: Database
DR: Dietary Record
EI: Energy Intake
FAO/INFOODS: Food and Agriculture Organization/International Network of Food Data Systems
FCDB: Food Composition Database
FCT: Food Composition Tables
FFQ: Food Frequency Questionnaire
FNDDS: Food and Nutrient Database for Dietary Studies
ICC: Intra-class correlation coefficients
IQR: Interquartile Range
Kcal: Kilocalorie
LOA: Limit of Agreement
NCDs: Non-communicable diseases
SCC: Spearman Correlation Coefficient
SD: Standard deviation
UAE: United Arab Emirates
UAEU: United Arab Emirates University
UK: United Kingdom
USDA SR DB: United States Department of Agriculture National Nutrient Database for Standard Reference
