# Supplementary material for: Validation of a quantitative web-based food frequency questionnaire to assess dietary intake in the adult Emirati population
Source: PLoS One. 2022 Jan 27;17(1):e0262150. doi: 10.1371/journal.pone.0262150 (PMC8794217; doi:10.1371/journal.pone.0262150)
Supplement: S1 Fig — (PDF) [file pone.0262150.s001.pdf]

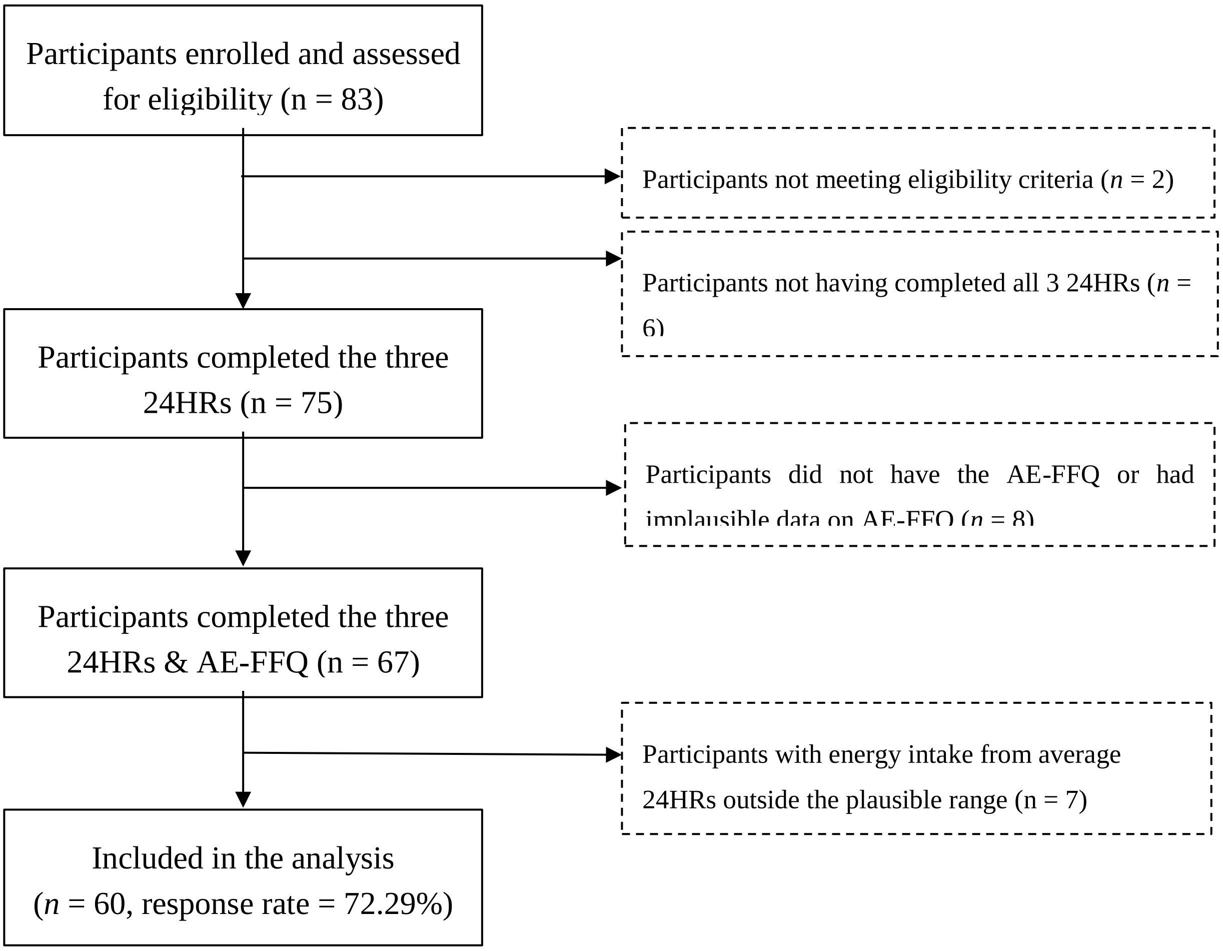

Figure S1. Flow of participants through the validation study

24HR = 24-hour dietary recall; AE-FFQ = Adult Emirati food frequency questionnaire.
