# Supplementary figures and images for: Validation of a quantitative web-based food frequency questionnaire to assess dietary intake in the adult Emirati population
Source: PLoS One. 2022 Jan 27;17(1):e0262150. doi: 10.1371/journal.pone.0262150 (PMC8794217; doi:10.1371/journal.pone.0262150)

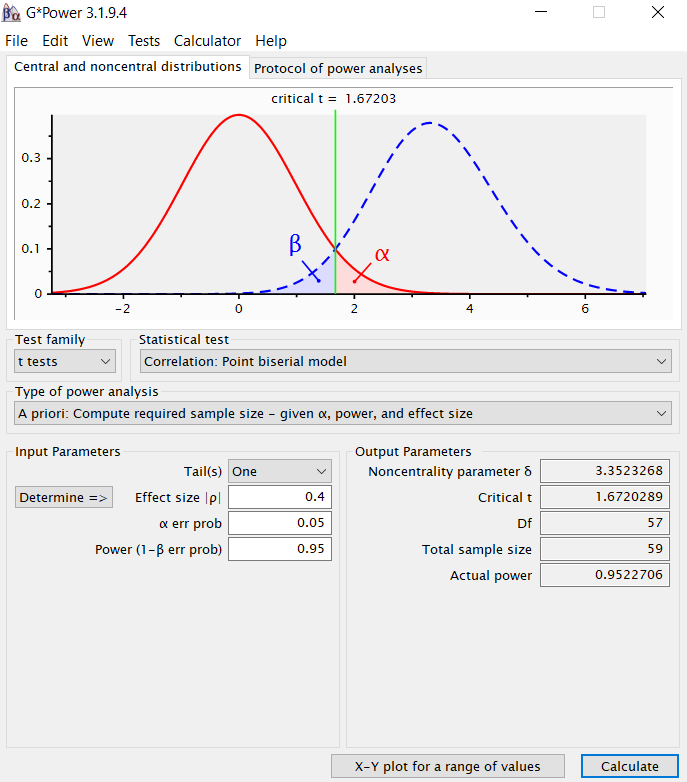

Supplement: S2 Fig — (PNG) [file pone.0262150.s002.png]
