# Supplementary material for: Validation of a quantitative web-based food frequency questionnaire to assess dietary intake in the adult Emirati population
Source: PLoS One. 2022 Jan 27;17(1):e0262150. doi: 10.1371/journal.pone.0262150 (PMC8794217; doi:10.1371/journal.pone.0262150)
Supplement: S1 Table — (PDF) [file pone.0262150.s003.pdf]

S-2. Table Categorization of the 31 food groups used for comparing food group intakes of the AE-FFQ by the three 24HRs

**Food group**	**Categories from the AE-FFQ or the three 24HRs**
Dairy drinks	Milk, Buttermilk, Laban up
Cheeses hard and spreadable	Cheddar, Mozzarella, Feta, Halloumi, Akkawi cheese, Labneh, triangle cheese, Kiri^{TM}
Yogurts	Plain and fruit yogurts
Rice and rice dishes	White rice, Biryani rice, Mandi rice, Machbous rice, rice from Sushi, Maqluba rice, rice in stuffed vegetables
Pasta & other cereal dishes (Oats)	Pasta dishes, lasagna, pasta with bechamel
White breads	Samoon bread, sliced bread, Rgag, paratha, buns. pizzas
Whole grains breads	Sliced whole grain, brown bread
legumes	Foul, baked beans, Lentils, lentils from Daal, cooked chickpeas, and chickpeas from Hummus
eggs	Egg fried, boiled, and Omelets
Red meat	All meat dishes excluding processed meats and sausages
Meat products	Processed meats; turkey salami or mortadella, sausages, shawarma meat, Beef, or chicken Hot Dog weiner or Frankfurter
Chicken	Chicken from all sources, chicken stewed, braised, with Skin, and without skin, Chicken tikka, roasted, fried, pan-fried, fried with skin, nuggets
Fish & Seafood	Fish and seafood from all sources cooked, baked, or fried, e.g. Red mullet fried, Cod flesh fried in batter, grilled seabass, grilled seabream, grilled Salmon, Mackerel, Tuna, Canned Tuna, Shrimp grilled, cooked or fried
Vegetables total	Vegetables from all sources, including from stews (Salona, Margoga, Thareed), in rice, or pasta dishes, sandwiches, and salads
Green leafy vegetables	Lettuce, Arugula, parsley from salads
Cruciferous vegetables	Cabbage, Broccoli, and cauliflower from mixed dishes
Red or yellow vegetables	Tomatoes, sweet potatoes, carrots cooked, and raw, pumpkin from any dish
Potatoes	From salads and mixed dishes, French fries not included

Other vegetables	All other vegetables not included in the above categories, e.g. Cucumber, eggplant, green beans, okra, peas, mushrooms etc.
Savory snacks	Fatayer, Pies, falafel, samosa, croissants, plain or with different fillings (cheese, thyme, or spinach)
Fruits	All fruits
Dried fruits	Dates and other dried fruits
Soft drinks, Including Energy Drinks	All soft drinks and energy drinks containing added sugar
Diet soft drinks	All soft drinks and energy drinks not containing added sugar
Fruit juices including smoothies	All commercial and fresh juices and smoothies (e.g. avocado smoothie)
sugar, syrups, jams, molasses, honey	Sugar or syrups added to beverages, jams, date molasses, and honey
French fries	French fries only
Sweet snacks	Biscuits (Oreo™, Digestive™, tea biscuit), cakes, muffins, doughnuts (glazed and plain), fruit pies, including Arabic sweets)
Sweets, candies, and chocolates	Candies, milk, and dark chocolates, chocolate bars
Chips	Potato chips and corn chips
Nuts & seeds	Mixed nuts, with, or without added Salt Added and pumpkin seeds

Foods groups in green depict foods evidenced as having protective effects in relation to NCDs [1].
Foods groups in red depict foods evidenced as having offensive effects in relation to NCDs [1].
24HR = 24-hour dietary recall; 3-d = 3-day; AE FFQ = food frequency–adult Emirati food frequency questionnaire.
